# Enhanced ISUP grade prediction in prostate cancer using multi-center radiomics data

**DOI:** 10.1007/s00261-025-04858-3

**Published:** 2025-03-06

**Authors:** Yuying Liu, Xueqing Han, Haohui Chen, Qirui Zhang

**Affiliations:** 1 Department of Computer Science and Technology, Nanjing University, Nanjing, China; 2 Kuang Yaming Honors School, Nanjing University, Nanjing, China; 3 Department of Radiology, Jinling Clinical Medical College, Nanjing University of Chinese Medicine, Nanjing, China; 4 Department of Neurology, Thomas Jefferson University, Philadelphia, USA

**Keywords:** Prostate cancer, ISUP grade, Radiomics, Machine learning, Bi-parametric MRI, Random forest model

## Abstract

**Background:**

To explore the predictive value of radiomics features extracted from anatomical ROIs in differentiating the International Society of Urological Pathology (ISUP) grading in prostate cancer patients.

**Methods:**

This study included 1,500 prostate cancer patients from a multi-center study. The peripheral zone (PZ) and central gland (CG, transition zone + central zone) of the prostate were segmented using deep learning algorithms and were defined as the regions of interest (ROI) in this study. A total of 12,918 image-based features were extracted from T2-weighted imaging (T2WI), apparent diffusion coefficient (ADC), and diffusion-weighted imaging (DWI) images of these two ROIs. Synthetic minority over-sampling technique (SMOTE) algorithm was used to address the class imbalance problem. Feature selection was performed using Pearson correlation analysis and random forest regression. A prediction model was built using the random forest classification algorithm. Kruskal-Wallis H test, ANOVA, and Chi-Square Test were used for statistical analysis.

**Results:**

A total of 20 ISUP grading-related features were selected, including 10 from the PZ ROI and 10 from the CG ROI. On the test set, the combined PZ + CG radiomics model exhibited better predictive performance, with an AUC of 0.928 (95% CI: 0.872, 0.966), compared to the PZ model alone (AUC: 0.838; 95% CI: 0.722, 0.920) and the CG model alone (AUC: 0.904; 95% CI: 0.851, 0.945).

**Conclusion:**

This study demonstrates that radiomic features extracted based on anatomical sub-region of the prostate can contribute to enhanced ISUP grade prediction. The combination of PZ + GG can provide more comprehensive information with improved accuracy. Further validation of this strategy in the future will enhance its prospects for improving decision-making in clinical settings.

## Introduction

Prostate cancer (PCa) is the most common malignant tumor in men, and its incidence rate is leading among male cancers [1]. It is predicted that by 2024, there will be 299,010 new diagnoses of PCa in the United States, and it is expected that 35,250 people will die from the disease [2]. The Gleason score (GS), a pathology standard for evaluating PCa aggressiveness, fails to differentiate between Gleason 3 + 4 = 7 and 4 + 3 = 7, a significant flaw that impacts patient care and has spurred efforts to improve the scoring system [3, 4]. In response to this need, the International Society of Urological Pathology (ISUP) introduced a new grading and grouping system for prognostic differences in PCa, updating the traditional GS scoring system. ISUP classifies PCa into five grades, with ISUP 1 (GS ≤ 3 + 3) being considered the lowest risk, while ISUP 5 (GS 9–10) is considered the highest risk [5]. The ISUP grading provides a more precise assessment of cancer risk for patients, so an accurate identification of the ISUP grading of PCa is critical for patient prognosis, risk stratification, and the development of individualized treatment plans. Misclassification of ISUP grading in PCa can lead to inappropriate treatment decisions, such as placing a patient with a higher-grade tumor on active surveillance when they may require definitive therapy, potentially resulting in disease progression and worse outcomes.

Magnetic resonance imaging (MRI) is an important imaging tool with significant clinical implications for the diagnosis and management of PCa. Multi-parametric MRI has high sensitivity and specificity for the detection of significant cancer using three imaging sequences, typically T2-weighted imaging (T2WI), diffusion-weighted imaging (DWI), and dynamic contrast-enhanced imaging (DCE) [6]. However, while multiparametric MRI requires the use of contrast agents and longer scan times, dual-parametric MRI (including T2WI and diffusion-weighted imaging) has the advantage of avoiding needles, avoiding injection of gadolinium enhancers, shorter scan times, and a lower price point, making it an increasingly important method of evaluation for staging and new diagnoses of PCa [7, 8]. The apparent diffusion coefficient (ADC) value is correlated with the histological grade of various malignant tumors. The DWI sequence and ADC map are considered to be the main tools for detecting and characterizing lesions through analysis [9–12]. However, the PCa risk scoring systems commonly used in imaging, such as PI-RADS [8], only assess the risk of PCa for clinical practice. It has also been found that patients with higher PI-RADS categories (4 or 5) have a greater risk of having clinically significant PCa [13, 14], but the direct correlation between PCa imaging and PCa pathologic grading to indicate severity and prognosis is underexplored.

Radiomics is an emerging field of research that explores the content related to diseases mainly by extracting a large amount of quantitative information from medical images, which can be used for various clinical applications such as disease diagnosis, prognosis assessment, and treatment response monitoring, and guide individualized treatment plans [15,–18]. Some researchers [19–22] have explored small data sets and studied the application of radiomics analysis based on [68Ga]Ga-PSMA-11 PET, [18 F] PSMA-1007 PET/CT and [68Ga] PSMA PET/CT in the prediction of International Society of Urological Pathology (ISUP) grading of PCa. Other efforts try to screen patients for clinically significant PCa using the mpMRI and radiomics [23–26]. Although these studies have shown encouraging results, the conclusions drawn may not be universally applicable when the dataset is expanded due to insufficient sample size (less than 500 cases in each), and the models have weak generalization capabilities.

This study aims to use a large-scale multi-center dataset to explore the potential value of radiomics features extracted based on anatomical ROIs for continuous ISUP grading of PCa, thereby achieving a more accurate image-pathology correlation.

## Methods

### Patients

We reviewed the PI-CAI: Public Training and Development Dataset [27], which comprises cases from more than 1,500 instances, sourced from multiple centers (Radboud University Medical Center, University Medical Center Groningen, Ziekenhuis Groep Twente). Each patient in the PI-CAI dataset includes three imaging sequences: axial T2WI, axial DWI, and axial ADC images. All patients may include sagittal and coronal T2WI sequences, and none include dynamic contrast enhancement (DCE) sequences. The sequences are aligned using RAS coordinates [28], and most of the images are aligned well after visual inspection. Manual registration is performed for a small number of images for which the alignment is poor.

### Segmentation mask

**Fig. 1:**
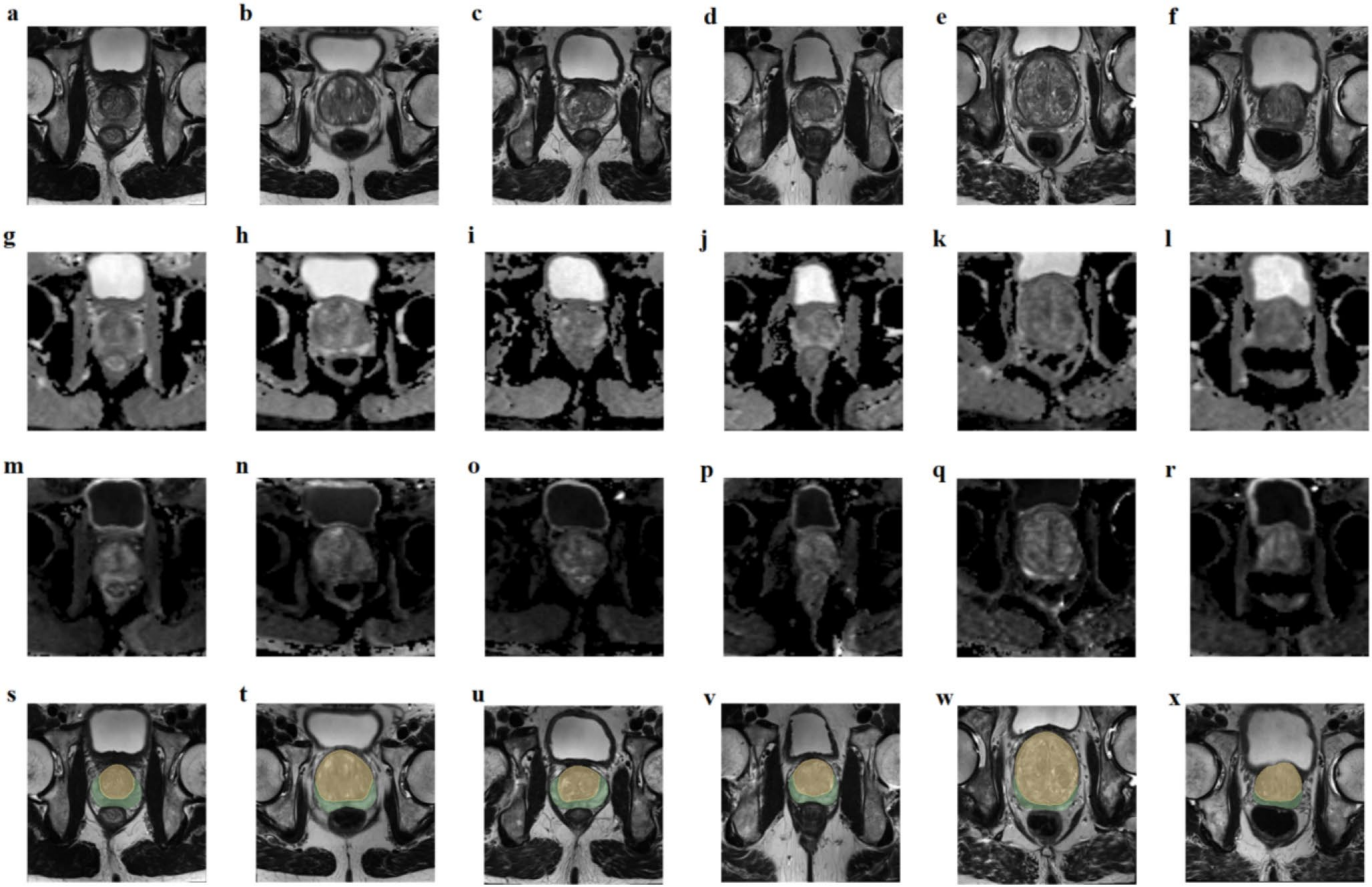
Examples T2WI, ADC and DWI images and masks on T2WI in PZ/CG: ISUP 0 of T2WI (**a**), ISUP 1 of T2WI (**b**), ISUP 2 of T2WI (**c**), ISUP 3 of T2WI (**d**), ISUP 4 of T2WI (**e**), ISUP 5 of T2WI (**f**), ISUP 0 of ADC (**g**), ISUP 1 of ADC (**h**), ISUP 2 of ADC (**i**), ISUP 3 of ADC (**j**), ISUP 4 of ADC (**k**), ISUP 5 of ADC (**l**), ISUP 0 of DWI (**m**), ISUP 1 of DWI (**n**), ISUP 2 of DWI (**o**), ISUP 3 of DWI (**p**), ISUP 4 of DWI (**q**), ISUP 5 of DWI (**r**), segmentation on T2WI of ISUP 0 (**s**), ISUP 1 (**t**), ISUP 2 (**u**), ISUP 3 (**v**), ISUP 4 (**w**), and ISUP 5 (**x**)

The segmentation labels of the peripheral zone (PZ) and central gland (CG, comprises the transition zone and the central zone) of the prostate were automatically performed using semi-supervised deep learning with T2WI modality [27]. Manual verification by one radiology expert was performed to ensure the quality of the anatomical mask, and did not find any obvious segmentation errors. The case examples of T2WI, ADC, and DWI images with ISUP = 0–5 and the mask on T2WI at PZ/CG are showed in Fig. 1. Since we used a publicly available dataset, no institutional review board or Health Insurance Portability and Accountability Act approval was required for this study.

### Feature extraction

**Fig. 2:**
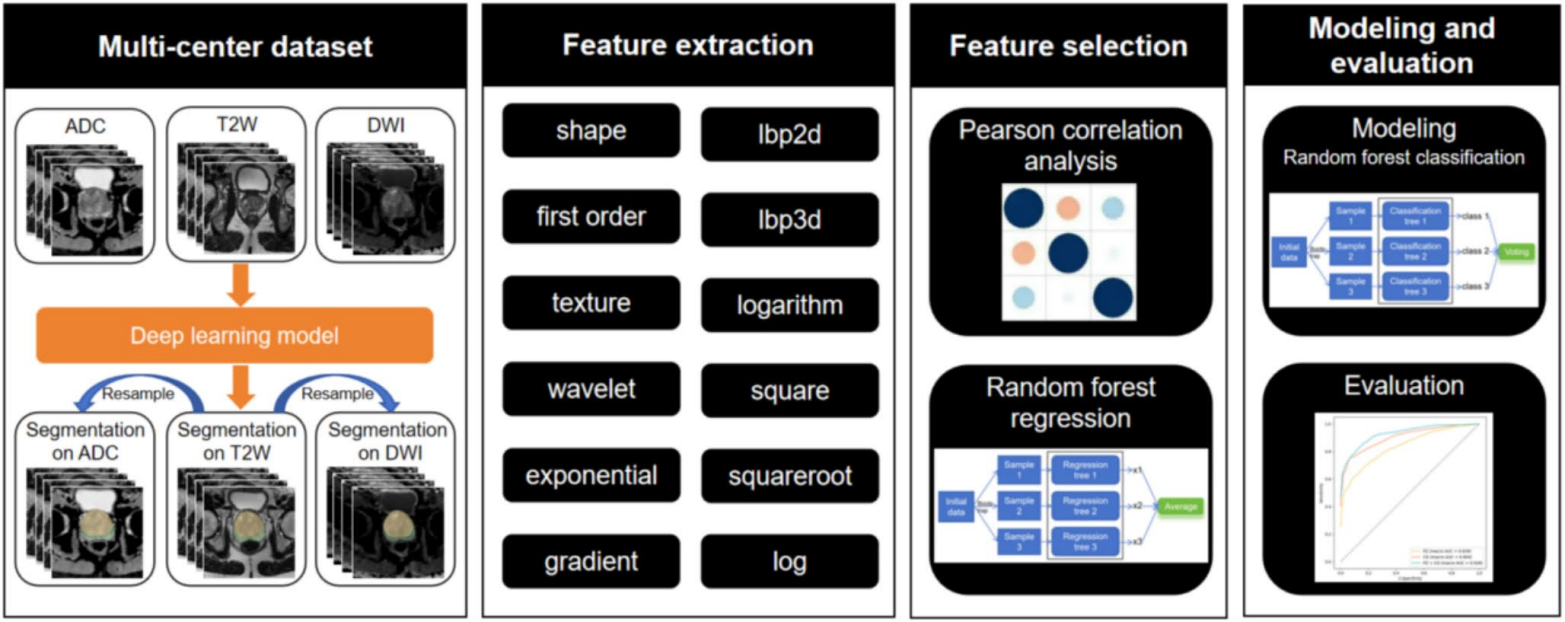
The study design of our research. It includes four steps: multi-center dataset, feature extraction, feature screening and modeling and evaluation

The study design of this research is showed in Fig. 2. For the 2 ROIs and 3 sequences, Pyradiomics (https://github.com/AIM-Harvard/pyradiomics) [29] was used to extract shape, first order, texture, wavelet, exponential, gradient, lbp2d, lbp3d, logarithm, square, square root and log features respectively, i.e., a total of 12,918 features were extracted for each patient. During the feature extraction process, set binWidth to 25, the resampling pixel spacing to 1 × 1 × 1, the resampling method to sitkBSpline, and the pad distance to 10.

### Dataset division and data enhancement

Random sampling was employed to allocate 15% of the data for the test set, with the remainder reserved for training. Baseline characteristics of the 1500 patients used in this study are summarized in Table 1. The data set is severely class-imbalanced between different ISUP grading, so data enhancement was performed in the training set using the SMOTE algorithm [30], while the test set retained its original distribution to ensure evaluation reliability. Since the SMOTE algorithm synthesizes new minority samples in the feature space to balance the dataset, thereby increasing the number of minority samples, and then improving the model’s prediction ability for the minority class, the SMOTE algorithm is also suitable for only smaller or less resourced dataset, i.e., the dataset size does not affect the reproducibility of the results.

**Table 1:** Characteristics of the ISUP = 0, ISUP = 1, ISUP = 2, ISUP = 3, ISUP = 4 and ISUP = 5 cohort.the classes are extremely imbalanced

Characteristics	0(*n* = 847)	1(*n* = 228)	2(*n* = 234)	3(*n* = 99)	4(*n* = 40)	5(*n* = 52)	*P*-value
Age (y)	64.115 ± 7.172	67.136 ± 6.476	67.491 ± 6.780	68.303 ± 6.119	67.100 ± 8.406	68.115 ± 7.633	0.000^a^
PSA (ng/mL²)	7.900(5.357-12.000)	7.800(5.582–11.550)	9.300(6.200–13.000)	12.000(7.375-19.000)	16.000(8.950–26.800)	14.500(8.075–28.975)	0.000^b^
Prostate volume (mL)	64.570(45.950-91.000)	49.000(35.000–68.000)	45.000(33.000–62.000)	49.000(36.250–62.750)	51.000(35.000-63.350)	54.500(38.750-68.868)	0.000^b^
PSAd (ng/mL²)	0.110(0.080–0.170)	0.150(0.110–0.223)	0.200(0.130–0.305)	0.230(0.155-0.400)	0.345(0.180–0.492)	0.365(0.207–0.490)	0.000^b^
center							0.000^c^
PCNN	161 (19.0%)	80 (35.1%)	74 (31.6%)	22 (22.2%)	7 (17.5%)	6 (11.5%)	
RUMC	475 (56.1%)	89 (39.0%)	121 (51.7%)	58 (58.6%)	27 (67.5%)	30 (57.7%)	
ZGT	211 (24.9%)	59 (25.9%)	39 (16.7%)	19 (19.2%)	6 (15.0%)	16 (30.8%)	

Notes: The results of the ANOVA analysis are displayed as mean ± standard deviation; the results of the Kruskal-Wallis H test are displayed as median (P25-P75); the results of the Chi-Square Test are displayed as number (proportion). PSA: Prostate-specific antigen level; PSAd: Prostate-specific antigen density. ^a^: ANOVA; ^b^: Kruskal-Wallis H test; ^c^Chi-Square Test

### Unstable feature exclusion

Due to differences in experimental conditions, operating procedures, sample sources, data processing methods, and other factors between research centers, some features extracted by raw imaging may be unstable. To further eliminate the impact of this factor on the experimental results, we used the intraclass correlation coefficient (ICC) [31] to eliminate features that are unstable between multiple centers, and only retained features with an ICC > = 0.75. Finally, 426 unstable features were eliminated, and 12,492 stable features were obtained.

### Feature selection

Pearson correlation analysis with a threshold of 0.95 was used for feature pre-screening, i.e., highly correlated features were screened out to avoid group effects. When features are highly correlated, the model may over-select one feature while ignoring other related features. This is called the “group effect”. Since least absolute shrinkage and selection operator (LASSO) regression [32] is sensitive to outliers and cannot handle nonlinear relationships, this study uses Random forest regression with a max depth of 10 for feature selection. Finally, based on the importance of the features, 10 features were selected separately for PZ and CG modeling in the next step. For the combined PZ + CG model, a total of 20 features were used, incorporating the 10 selected features from PZ model and the 10 selected features from CG model. The feature selection process was performed only on the training set.

### Model construction and evaluation

Three radiomics models were built based on different anatomical ROIs, namely the PZ model, CG model, and PZ + CG model. All models were built using a random forest classifier, and the optimal hyperparameters were determined using grid search. Model evaluation used multiple indicators such as AUC-ROC and AUC-PR curves, AUC and AUPRC values, accuracy, recall, precision, and kappa coefficient.

### Statistical analysis

The Python 3.7.9 (http://www.python.org) software was used to perform statistical analysis of the clinical characteristics. Since ANOVA compares the differences between multiple groups in one or more dependent variables, ANOVA was used for the age variable between different groups [33]; for variables such as prostate-specific antigen level, prostate-specific antigen density, and prostate volume, since they are non-normally distributed continuous variables, the Kruskal-Wallis H test [34] was used to compare the differences between groups; for variables as the center, since the central data are unordered and independent, the Chi-Square Test was used. A P value of less than 0.05 is used as the criterion for judging whether the statistical result is significant.

## Results

### Baseline information

Baseline characteristics of the 1500 patients used in this study are summarized in Table 1. According to the ISUP score, there are six different statistical groups: ISUP = 0, ISUP = 1, ISUP = 2, ISUP = 3, ISUP = 4, and ISUP = 5. We found with increasing age, clinical variables such as prostate-specific antigen level, prostate-specific antigen density, and prostate volume all show an increasing trend.

### Characteristics selected by different anatomical ROI models

**Fig. 3:**
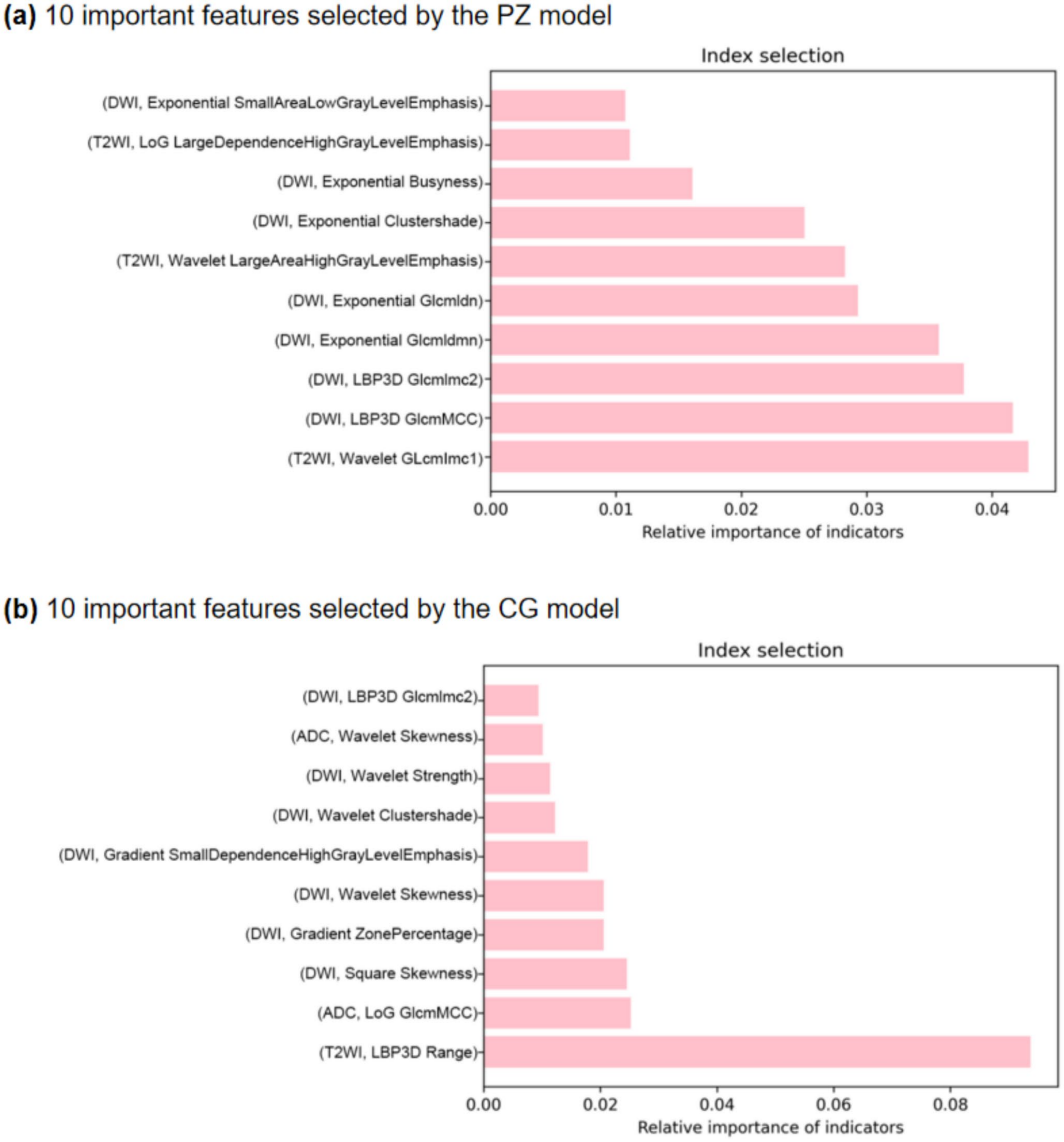
A histogram of the 10 important features and the importance of the features selected by the PZ model (**a**), CG model (**b**)

The 10 important features selected by the PZ model and the CG model using the random forest regressor and the feature importance histogram are showed in Fig. 3 (a) and (b), respectively.

In the PZ model, 3 features are derived from T2WI, and 7 features are obtained from DWI, as illustrated in Fig. 3 (a). Wavelet GLcmImc1 features in T2WI have the largest weight, reflecting the heterogeneity and spatial dependence within the tumor, while high-grade prostate cancer (ISUP 3–5) generally exhibits higher heterogeneity and aggressiveness. The LBP3D and GLCM features (e.g., GlcmMCC and Glcmlmc2) in the DWI sequence further capture the three-dimensional spatial heterogeneity and structural complexity of the tumor, These features play an important role in distinguishing between high-grade and low-grade tumors. Exponential transformed GLCM features (e.g., Glcmldmn and Glcmldn) may highlight the dependence and non-uniform changes in the microstructure of tumors in DWI sequences, further assisting ISUP grading. In addition, the Wavelet Large AreaHighGrayLevelEmphasis and LoG LargeDependenceHighGrayLevelEmphasis features of T2WI reflect the large-area distribution and spatial dependence of high gray value regions, respectively, and may be related to necrosis or hemorrhagic areas of high-grade tumors. The Exponential Clustershade and Busyness features of DWI capture the local contrast and texture complexity of the tumor.

In the CG model, 1 feature is derived from T2WI, 2 features are obtained from ADC maps, and 7 features are extracted from DWI, as shown in Fig. 3(b). The feature with the highest weight is the LBP3D Range feature of the T2WI sequence. The range of intensity changes through local binary patterns (LBP3D) reflects the heterogeneity of the tumor region, and high-grade tumors usually exhibit higher heterogeneity. (ADC, LoG GlcmMCC) combines Laplacian of Gaussian filtering (LoG) and the maximum correlation coefficient of the gray co-occurrence matrix (GlcmMCC) to describe the complexity of the internal structure of the tumor. High-grade tumors usually have more complex microstructures. (DWI, Square Skewness) and (DWI, Wavelet Skewness) both analyze the asymmetry of the intensity distribution in the tumor region by means of skewness, which may be related to necrotic or hemorrhagic areas within the tumor. (DWI, Gradient Zone%) and (DWI, Gradient SmallDependenceHighGrayLevelEmphasis) are both based on gradient analysis. The former describes the sharpness of the tumor edge, while the latter reflects the local dependence of high gray level pixels. Together, they suggest that high-grade tumors may have more irregular edges and more high-signal areas. (DWI, Wavelet Clustershade) and (DWI, Wavelet Strength) both analyze the texture and signal intensity changes in the tumor area using wavelet transform. The former describes the complexity of the texture, while the latter reflects the multi-scale signal intensity distribution. High-grade tumors usually show more complex textures and significant multi-scale signal changes.

### Predictive ability of different anatomical ROI models for ISUP grading tasks

**Fig. 4:**
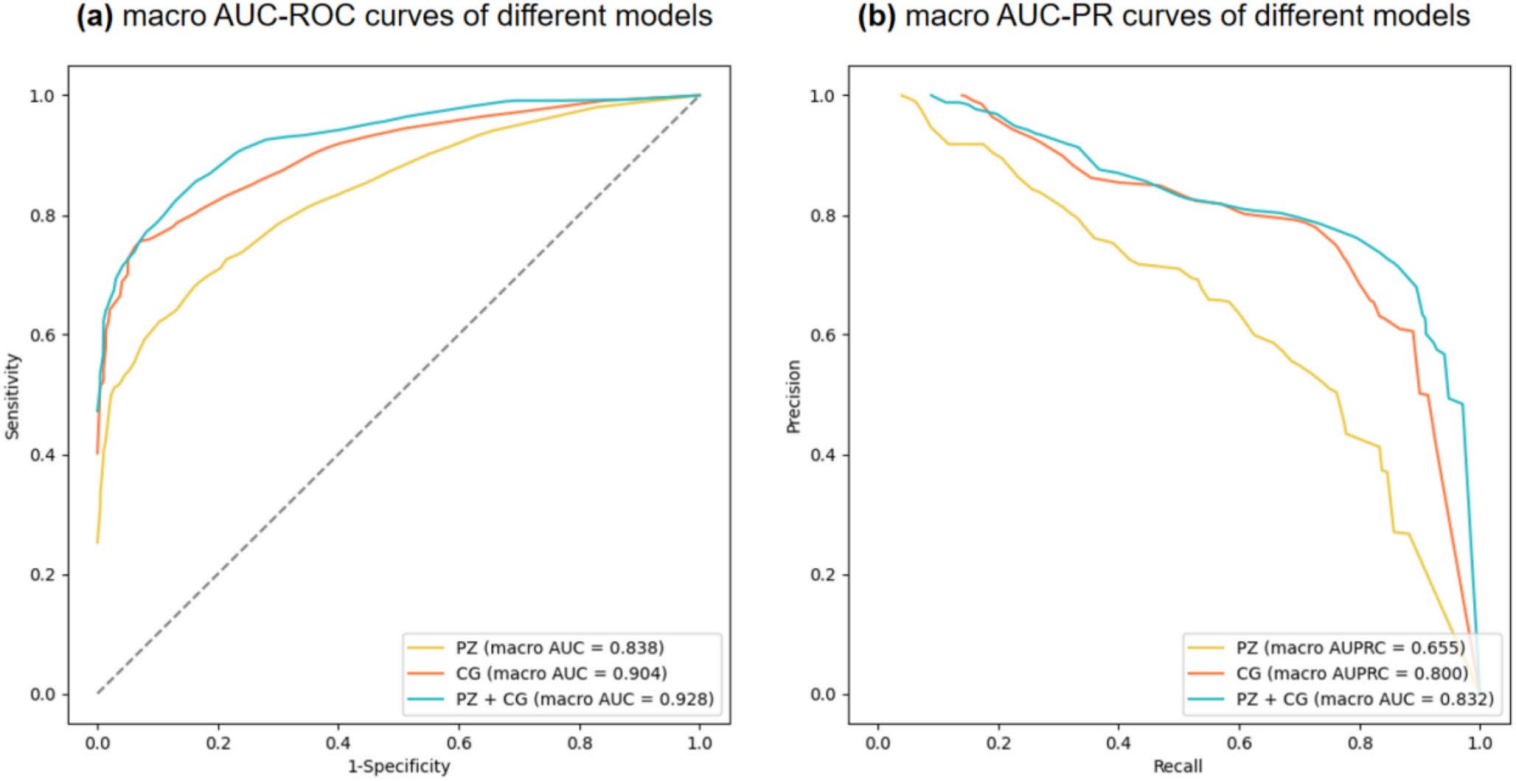
(**a**) The macro AUC-ROC curves for different anatomical ROI models. (**b**) The macro AUC-PR curves for different models. Among them, the PZ + CG After model has the best AUC-ROC and AUC-PR curve performance

**Fig. 5:**
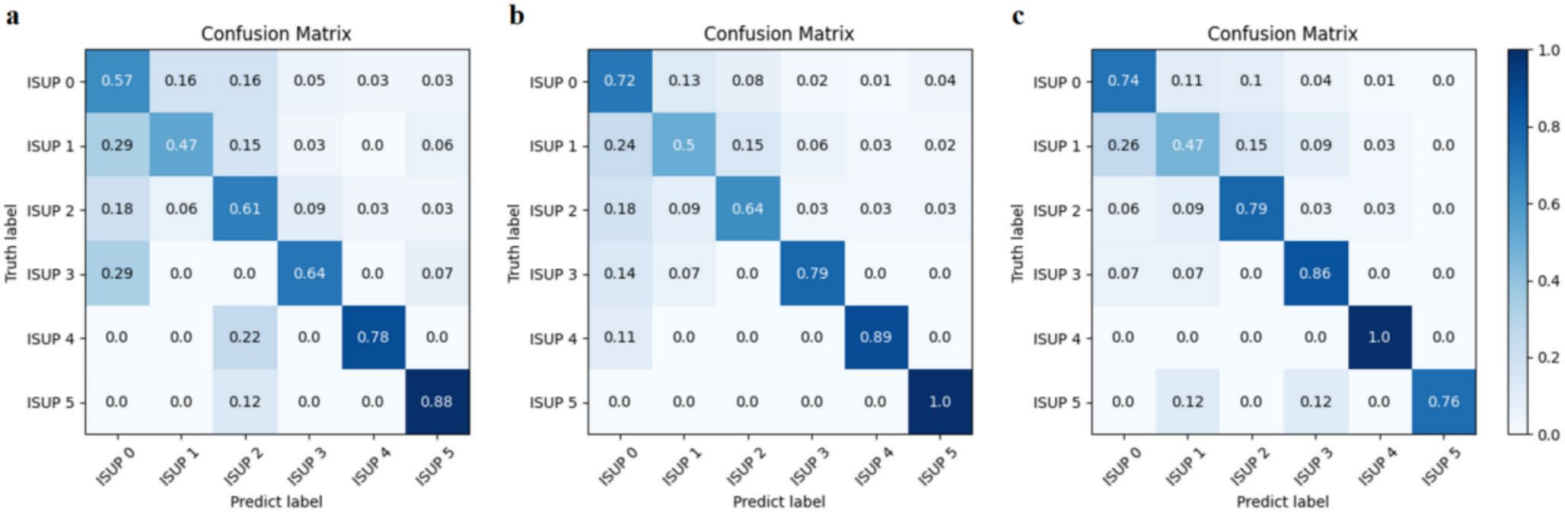
Classification confusion matrix of the three anatomical ROI models. PZ model confusion matrix (**a**), CG model confusion matrix (**b**), PZ + CG combined model confusion matrix (**c**). The CG model has the best performance for predicting ISUP 1 and ISUP 5, while the PZ + CG model mainly improves the prediction results for ISUP 2–4

The results of different anatomical ROI models for ISUP grading tasks are showed in Table 2. All models achieved relatively good AUCs on the training set. On the test set, the AUC of the PZ model was 0.838 (95%CI: 0.722, 0.920), and the AUC of the CG model was 0.904 (95%CI: 0.851, 0.945), and the PZ + CG model achieved the best performance (AUC: 0.928; 95%CI: 0.872, 0.966). The macro AUC-ROC curves and macro AUC-PR curves are shown in Fig. 4. The confusion matrix of different models are shown in Fig. 5. From Fig. 5, we find that the CG model has the best prediction effect on ISUP 1 and ISUP 5, especially ISUP 5, where the confusion matrix result reaches 1. The PZ + CG model mainly improved the prediction results of ISUP 2–4, corresponding to the intermediate and high grades of PCa The results show that the PZ + CG model can improve the accuracy of the ISUP grading of prostate cancer to a certain extent. However, when the number of cases in a category is small, the performance will decrease slightly.

**Table 2:** The results of different anatomical ROI models on the ISUP grading task. The PZ + CG model performs best on the test set

	Model	AUC (95%CI)	AUPRC (95%CI)	Accuracy	Recall	Precision	Kappa
Training set	PZ	1.000 (1.000, 1.000)	1.000 (1.000, 1.000)	0.999	0.999	0.999	0.999
	CG	1.000 (1.000, 1.000)	1.000 (1.000, 1.000)	1.000	1.000	1.000	0.999
	PZ + CG	1.000 (1.000, 1.000)	1.000 (1.000, 1.000)	1.000	1.000	1.000	1.000
Test set	PZ	0.838 (0.722, 0.920)	0.655 (0.421, 0.839)	0.581	0.656	0.518	0.403
	CG	0.904 (0.851, 0.945)	0.800 (0.607, 0.881)	0.696	0.755	0.631	0.545
	PZ + CG	**0.928 (0.872**,** 0.966)**	**0.832 (0.676**,** 0.933)**	**0.722**	**0.767**	**0.681**	**0.587**

AUC = area under the curve; AUPRC = area under the precision-recall curve; PZ = peripheral zone; CG = central gland;

## Discussion

In this paper, we used a large multi-center prostate data set to explore the predictive performance of anatomical ROI-based radiomics analysis for ISUP grading of PCa. The results showed that the combined model (PZ + CG) using the random forest classification algorithm performed better than the individual PZ and CG models on the test set.

Through biparametric MRI, radiomics, combined with a random forest algorithm, we have established a good image-pathology correlation of the PCa. This once again proves the ability of radiomics to reflect pathological grading in urological tumors. Previous studies have also confirmed that texture features or radiomics can predict the ISUP grading of renal cell carcinoma [35–37]. In clinical settings, first, pre-determining the severity of a patient’s prostate cancer is critical for deciding the best treatment plan. However, there is still some uncertainty, which may lead to the misclassification of intermediate-risk patients, which may result in excessive or unnecessary treatment. Our research results show that radiomics feature prediction can provide a useful tool to help clinicians make more accurate diagnoses, thereby reducing unnecessary biopsy procedures and prevent misclassification of intermediate-risk patients. Second, the difference in performance between ISUP grades (e.g., grades 1,2 and 3–5) is critical to treatment decisions for patients. In particular, for patients with low-risk PCa (ISUP 1), a more conservative treatment strategy can be used due to the lower degree of malignancy, such as active surveillance [38, 39]; as for patients with intermediate-risk PCa (ISUP 2), active surveillance and brachytherapy are also two effective treatment options for favorable PCa, and partial gland ablation is a wise choice for carefully selected PCa [40]; while for patients with ISUP grade 3–5, a more aggressive treatment strategy such as surgery or radiation therapy may be required, possibly in combination with endocrine therapy or chemotherapy, due to the higher malignancy of the tumor [41, 42]. Therefore, accurate ISUP grade assessment is critical for developing an individualized treatment plan, which can help prostate cancer patients achieve better treatment outcomes and a higher quality of life.

But, previous efforts in imaging-pathological correlation in quantitative analysis of PCa [19–22], the present study achieves graded controls at fine grains instead of binary (clinically significant PCa / non-clinically significant) classification. However, all of these models require manual segmentation of the PCa region, which leads to a limited potential for clinical utility considering that these strategy requires the physician to segment the lesion layer by layer, not to mention the fact that it is more difficult to determine the boundaries of PCa compared to other tumors. And it is for the same reason that automated PCa lesion segmentation based on artificial intelligence remains challenging to provide lesion segmentation to radiomics [43].

Our radiomics strategy based on the anatomical zones of the prostate has once again verified that the global characteristics of the prostate are significantly correlated with the pathological grading of prostate cancer [24, 44, 45]. We found that radiomics analysis based on biparametric MRI can effectively predict the ISUP score of prostate cancer, providing an important basis for personalized treatment plans. In real clinical practice, this method can improve the diagnostic workflow for prostate cancer, especially in terms of risk assessment before biopsy. Specifically, it can help clinicians determine whether patients need immediate surgery or further imaging tests, potentially reducing the rate of unnecessary biopsies. In addition, for ambiguous cases, the method provides a more accurate ISUP grade, which optimizes the patient’s treatment strategy. By using a random forest model to comprehensively analyze imaging features, clinicians can not only guide individualized treatment, but also continuously monitor patients’ imaging changes during follow-up to detect tumors that may recur early, thereby further improving patient survival rates.

A total of 12,918 image-based features were extracted from T2WI, ADC, and DWI images of PZ and CG ROIs. In T2WI images, the prostate is typically visualized as two distinct zones: the CG, which includes the transition zone and central zone, and the peripheral zone (PZ). Our focus on segmenting the PZ and CG aligns with current clinical practices and enables more stable and reliable prostate segmentation [46]. The PZ is of particular clinical significance, as it is the site where over 70% of prostate cancers (PCas) originate, making it a primary target for biopsy. In contrast, the incidence of PCas in the CG region (including the TZ and CZ) is less than 30% [47, 48]. Since LASSO regression is sensitive to outliers and cannot effectively model nonlinear relationships, this study employed a random forest regressor for feature selection. From this analysis, we identified 20 features associated with the ISUP grading of prostate cancer (PCa), 14 of them are from DWI, which is consistent with PI-RADS recommends DWI as the imaging sequence of choice for evaluating PZ lesions and determining the malignancy of transition zone lesions [8].

Despite the significance of the findings of this study, we must also acknowledge the challenges faced. First, the variability between multiple centers may affect the consistency and reliability of the data. Second, the standardization of imaging protocols is essential to ensure the reproducibility of results between different laboratories. Finally, the computational infrastructure required to implement the random forest model may limit its widespread application.

This study had some limitations. First, for some patients, clinical features such as prostate-specific antigen level, and prostate-specific antigen density could not be included in the prediction model, but they are commonly used by clinicians to assess the aggressiveness of PCa [49, 50]. Second, while the diagnostic performance of the combined model PZ + CG improved at ISUP grades 2–4, it slightly decreased at ISUP grades 1 and 5. This discrepancy may be attributed to the imbalanced distribution of categories within the multicenter dataset. Although this study utilized multi-center data, it was derived from a single dataset, which may introduce potential bias. To enhance the robustness and generalizability of the findings, future studies should incorporate external validation using independent datasets from different populations or institutions. Additionally, employing cross-validation techniques during model development could help mitigate overfitting and provide a more reliable assessment of the model’s performance across diverse patient cohorts. Prospective studies based on these strategies are warranted to further validate the model’s robustness and clinical applicability.

## Conclusion

In conclusion, we demonstrated the promising performance of anatomical ROI-based radiomics models in predicting the ISUP grade of PCa using a large multi-center prostate cancer dataset, which may improve diagnosis and treatment workflows for prostate cancer.

## Data Availability

https://pi-cai.grand-challenge.org/DATA/.

## References

[1] Benson 3rd AB, Abrams TA, Ben-Josef E et al. (2009) NCCN clinical practice guidelines in oncology: hepatobiliary cancers. Journal of the National Comprehensive Cancer Network: JNCCN 7:350–391 19406039 10.6004/jnccn.2009.0027 PMC4461147

[2] Siegel RL, Giaquinto AN, Jemal A (2024) Cancer statistics, 2024. CA: a cancer journal for clinicians 74:12–49 38230766 10.3322/caac.21820

[3] Zhuang H, Chatterjee A, Fan X et al. (2023) A radiomics based method for prediction of prostate cancer Gleason score using enlarged region of interest. BMC Medical Imaging 23:205 38066434 10.1186/s12880-023-01167-3 PMC10709874

[4] Egevad L, Delahunt B, Srigley JR et al. (2016) International Society of Urological Pathology (ISUP) grading of prostate cancer–An ISUP consensus on contemporary grading. In:Wiley Online Library, p 433–435 10.1111/apm.12533 27150257

[5] Samaratunga H, Delahunt B, Yaxley J et al. (2016) From Gleason to International Society of Urological Pathology (ISUP) grading of prostate cancer. Scandinavian journal of urology 50:325–329 27415753 10.1080/21681805.2016.1201858

[6] Bass E, Pantovic A, Connor M et al. (2021) A systematic review and meta-analysis of the diagnostic accuracy of biparametric prostate MRI for prostate cancer in men at risk. Prostate Cancer and Prostatic Diseases 24:596–611 33219368 10.1038/s41391-020-00298-w

[7] Zhang Y, Li Z, Gao C et al. (2024) Radiomic nomogram based on bi-parametric magnetic resonance imaging to predict the International Society of Urological Pathology grading ≥ 3 prostate cancer: a multicenter study. Clinical Radiology 10.1016/j.crad.2024.04.011 38763807

[8] Turkbey B, Rosenkrantz AB, Haider MA et al. (2019) Prostate imaging reporting and data system version 2.1: 2019 update of prostate imaging reporting and data system version 2. European urology 76:340–351 30898406 10.1016/j.eururo.2019.02.033

[9] Sugahara T, Korogi Y, Kochi M et al. (1999) Usefulness of diffusion-weighted MRI with echo‐planar technique in the evaluation of cellularity in gliomas. Journal of Magnetic Resonance Imaging: An Official Journal of the International Society for Magnetic Resonance in Medicine 9:53–60 10.1002/(sici)1522-2586(199901)9:1<53::aid-jmri7>3.0.co;2-2 10030650

[10] Yabuuchi H, Soeda H, Matsuo Y et al. (2006) Phyllodes tumor of the breast: correlation between MR findings and histologic grade. Radiology 241:702–709 17032912 10.1148/radiol.2413051470

[11] Higano S, Yun X, Kumabe T et al. (2006) Malignant astrocytic tumors: clinical importance of apparent diffusion coefficient in prediction of grade and prognosis. Radiology 241:839–846 17032910 10.1148/radiol.2413051276

[12] Itou Y, Nakanishi K, Narumi Y et al. (2011) Clinical utility of apparent diffusion coefficient (ADC) values in patients with prostate cancer: can ADC values contribute to assess the aggressiveness of prostate cancer? Journal of Magnetic Resonance Imaging 33:167–172 21182135 10.1002/jmri.22317

[13] Dekalo S, Mazliah O, Barkai E et al. (2024) MRI-based PI-RADS score predicts ISUP upgrading and adverse pathology at radical prostatectomy in men with biopsy ISUP 1 prostate cancer. The Canadian Journal of Urology 31:11955–11962 39217520

[14] Hongo F, Nakanouchi T, Nakamura J et al. (1998) Predictability of Gleason score and reduction time (tau) of prostatic volume after castration for the prognosis of prostatic cancer. Nihon Hinyokika Gakkai zasshi. The Japanese Journal of Urology 89:871–875 9866376 10.5980/jpnjurol1989.89.871

[15] Jin J, Jiang Y, Zhao Y-L et al. (2024) Radiomics-based machine learning to predict the recurrence of hepatocellular carcinoma: a systematic review and meta-analysis. Academic Radiology 31:467–479 37867018 10.1016/j.acra.2023.09.008

[16] Ye J-Y, Fang P, Peng Z-P et al. (2024) A radiomics-based interpretable model to predict the pathological grade of pancreatic neuroendocrine tumors. European radiology 34:1994–2005 37658884 10.1007/s00330-023-10186-1 PMC10873440

[17] Xia T, Zhao B, Li B et al. (2024) MRI-based radiomics and deep learning in biological characteristics and prognosis of hepatocellular carcinoma: Opportunities and challenges. Journal of Magnetic Resonance Imaging 59:767–783 37647155 10.1002/jmri.28982

[18] Tafuri B, Milella G, Filardi M et al. (2024) Machine learning-based radiomics for amyotrophic lateral sclerosis diagnosis. Expert Systems with Applications 240:122585

[19] Ghezzo S, Mapelli P, Bezzi C et al. (2023) Role of [68Ga] Ga-PSMA-11 PET radiomics to predict post-surgical ISUP grade in primary prostate cancer. European Journal of Nuclear Medicine and Molecular Imaging 50:2548–2560 36933074 10.1007/s00259-023-06187-3

[20] Feliciani G, Celli M, Ferroni F et al. (2022) Radiomics analysis on [68Ga] Ga-PSMA-11 PET and MRI-ADC for the prediction of prostate cancer ISUP grades: preliminary results of the BIOPSTAGE trial. Cancers 14:1888 35454793 10.3390/cancers14081888 PMC9028386

[21] Yang F, Wang C, Shen J et al. (2024) End-to-end [18F] PSMA-1007 PET/CT radiomics-based pipeline for predicting ISUP grade group in prostate cancer. Abdominal Radiology. 2024 Sep 30. 10.1007/s00261-024-04601-4 39349643

[22] Zhang H, Tang Y, Qi L et al. (2024) MP74-15 MACHINE LEARNING-BASED CONSTRUCTION AND VALIDATION OF A 68GA-PSMA PET/CT RADIOMICS MODEL FOR PREDICTING ISUP GRADING IN PROSTATE CANCER. Journal of Urology 211:e1199 10.1007/s00259-025-07412-x 40553115

[23] Zhang H, Li X, Zhang Y et al. (2021) Diagnostic nomogram based on intralesional and perilesional radiomics features and clinical factors of clinically significant prostate cancer. Journal of Magnetic Resonance Imaging 53:1550–1558 33851471 10.1002/jmri.27486

[24] Krauss W, Frey J, Heydorn Lagerlöf J et al. (2024) Radiomics from multisite MRI and clinical data to predict clinically significant prostate cancer. Acta Radiologica 65:307–317 38115809 10.1177/02841851231216555 PMC10964389

[25] Li T, Sun L, Li Q et al. (2022) Development and validation of a radiomics nomogram for predicting clinically significant prostate cancer in PI-RADS 3 lesions. Frontiers in oncology 11:825429 35155214 10.3389/fonc.2021.825429 PMC8825569

[26] Chen Z-L, Huang Z-C, Lin S-S et al. (2024) Clinical value of a radiomics model based on machine learning for the prediction of prostate cancer. Journal of International Medical Research 52:03000605241275338 39370971 10.1177/03000605241275338 PMC11459546

[27] Saha A, Bosma JS, Twilt JJ et al. (2024) Artificial intelligence and radiologists in prostate cancer detection on MRI (PI-CAI): an international, paired, non-inferiority, confirmatory study. The Lancet Oncology 10.1016/S1470-2045(24)00220-1 PMC11587881 38876123

[28] Kikinis R, Pieper SD, Vosburgh KG (2013) 3D Slicer: a platform for subject-specific image analysis, visualization, and clinical support. In: Jolesz, F. (eds) Intraoperative imaging and image-guided therapy. Springer, p 277–289

[29] Van Griethuysen JJ, Fedorov A, Parmar C et al. (2017) Computational radiomics system to decode the radiographic phenotype. Cancer research 77:e104-e107 29092951 10.1158/0008-5472.CAN-17-0339 PMC5672828

[30] Chawla NV, Bowyer KW, Hall LO et al. (2002) SMOTE: synthetic minority over-sampling technique. Journal of artificial intelligence research 16:321–357

[31] Xue C, Yuan J, Lo GG et al. (2021) Radiomics feature reliability assessed by intraclass correlation coefficient: a systematic review. Quantitative imaging in medicine and surgery 11:4431 34603997 10.21037/qims-21-86 PMC8408801

[32] Ranstam J, Cook JA (2018) LASSO regression. Journal of British Surgery 105:1348–1348

[33] St L, Wold S (1989) Analysis of variance (ANOVA). Chemometrics and intelligent laboratory systems 6:259–272

[34] Vargha A, Delaney HD (1998) The Kruskal-Wallis test and stochastic homogeneity. Journal of Educational and behavioral Statistics 23:170–192

[35] Cui E, Li Z, Ma C et al. (2020) Predicting the ISUP grade of clear cell renal cell carcinoma with multiparametric MR and multiphase CT radiomics. European radiology 30:2912–2921 32002635 10.1007/s00330-019-06601-1

[36] Xv Y, Lv F, Guo H et al. (2021) A CT-based radiomics nomogram integrated with clinic-radiological features for preoperatively predicting WHO/ISUP grade of clear cell renal cell carcinoma. Frontiers in Oncology 11:712554 34926241 10.3389/fonc.2021.712554 PMC8677659

[37] Liu X, Han X, Wang X et al. (2025) Development and validation of a CT based radiomics nomogram for preoperative prediction of ISUP/WHO grading in renal clear cell carcinoma. Abdominal Radiology 50:1228–1239 10.1007/s00261-024-04576-2 39311950

[38] Moschini M, Carroll PR, Eggener SE et al. (2017) Low-risk prostate cancer: identification, management, and outcomes. European Urology 72:238–249 28318726 10.1016/j.eururo.2017.03.009

[39] Briganti A, Fossati N, Catto JW et al. (2018) Active surveillance for low-risk prostate cancer: the European Association of Urology position in 2018. European urology 74:357–368 29937198 10.1016/j.eururo.2018.06.008

[40] Preisser F, Cooperberg MR, Crook J et al. (2020) Intermediate-risk prostate cancer: stratification and management. European Urology Oncology 3:270–280 32303478 10.1016/j.euo.2020.03.002

[41] Chang AJ, Autio KA, Roach Iii M et al. (2014) High-risk prostate cancer—classification and therapy. Nature reviews Clinical oncology 11:308–323 24840073 10.1038/nrclinonc.2014.68 PMC4508854

[42] Stock RG, Cahlon O, Cesaretti JA et al. (2004) Combined modality treatment in the management of high-risk prostate cancer. International Journal of Radiation Oncology* Biology* Physics 59:1352–1359 15275720 10.1016/j.ijrobp.2004.01.023

[43] Arif M, Schoots IG, Castillo Tovar J et al. (2020) Clinically significant prostate cancer detection and segmentation in low-risk patients using a convolutional neural network on multi-parametric MRI. European radiology 30:6582–6592 32594208 10.1007/s00330-020-07008-z PMC7599141

[44] Wu Y, Tian J, Ma F et al. (2024) Can dynamic contrast-enhanced MR imaging based on radiomics improve the diagnostic efficiency of clinically significant prostate cancer? Br. J. Hosp. Med. 85:1–13 10.12968/hmed.2024.0131 39078901

[45] Gong L, Xu M, Fang M et al. (2022) The potential of prostate gland radiomic features in identifying the Gleason score. Computers in biology and medicine 144:105318 35245698 10.1016/j.compbiomed.2022.105318

[46] Qiu W, Yuan J, Ukwatta E et al. (2014) Dual optimization based prostate zonal segmentation in 3D MR images. Medical image analysis 18:660–673 24721776 10.1016/j.media.2014.02.009

[47] Khan Z, Yahya N, Alsaih K et al. (2021) Recent automatic segmentation algorithms of MRI prostate regions: a review. IEEE Access 9:97878–97905

[48] Duran A, Dussert G, Rouvière O et al. (2022) ProstAttention-Net: A deep attention model for prostate cancer segmentation by aggressiveness in MRI scans. Medical Image Analysis 77:102347 35085952 10.1016/j.media.2021.102347

[49] Perdana NR, Mochtar CA, Umbas R et al. (2017) The risk factors of prostate cancer and its prevention: a literature review. Acta medica indonesiana 48:228–238 27840359

[50] Castillo T JM, Arif M, Starmans MP et al. (2021) Classification of clinically significant prostate cancer on multi-parametric MRI: a validation study comparing deep learning and radiomics. Cancers 14:12 35008177 10.3390/cancers14010012 PMC8749796

